# Ovulation induction versus artificial cycles in polycystic ovary syndrome: higher live birth rate but elevated gestational diabetes risk

**DOI:** 10.1186/s12884-026-09640-x

**Published:** 2026-07-14

**Authors:** Jinlin Xie, Juanzi Shi, Xitong Liu

**Affiliations:** The Assisted Reproduction Center, Northwest Women’s and Children’s Hospital, Xi’an, China

**Keywords:** Gestational diabetes mellitus, Large for gestational age, Live birth, Polycystic ovary syndrome

## Abstract

**Background:**

Polycystic ovary syndrome (PCOS) is a common endocrine disorder associated with infertility, and frozen-thawed embryo transfer (FET) has become a mainstream assisted reproductive technology for PCOS patients. Two primary endometrial preparation protocols are widely used for FET: ovulation induction cycles and artificial cycles (AC). However, the comparative clinical efficacy, maternal outcomes, and perinatal outcomes of these two protocols in PCOS women remain inconsistent in existing literature, highlighting the need for large-scale cohort studies to provide evidence-based guidance. This study aimed to investigate whether clinical, maternal, and perinatal outcomes following FET differ between ovulation induction cycles and artificial cycles in women with PCOS.

**Methods:**

This was a retrospective cohort study conducted at a public fertility center in China. A total of 3,255 PCOS women who underwent FET between January 2018 and December 2023 were included. After propensity score matching (1:1), 530 patients in the ovulation induction group were compared with 530 patients in the artificial cycle group. The primary outcome was live birth rate. Secondary outcomes included clinical pregnancy, miscarriage, maternal outcomes, and perinatal outcomes.

**Results:**

After PSM, the ovulation induction group exhibited a significantly higher live birth rate than the artificial cycle group (57.5% vs. 51.3%; odds ratio [OR] 1.3, 95% confidence interval [CI] 1.0–1.7). The ovulation induction group also had a lower miscarriage rate (11.1% vs. 15.8%; OR 0.7, 95% CI 0.4–0.9). Regarding maternal outcomes, the ovulation induction group was associated with a significantly elevated risk of gestational diabetes mellitus (GDM) (17.6% vs. 9.0%; OR 2.2, 95% CI 1.4–3.4; *P* < 0.01). In singleton pregnancies, the ovulation induction group showed a reduced risk of large for gestational age (LGA) infants compared to the artificial cycle group (22.7% vs. 36.9%; OR 0.5, 95% CI 0.3–0.7; *P* < 0.01).

**Conclusions:**

Within the limitations of a retrospective design and single-center setting, this study shows that in PCOS women undergoing FET, ovulation induction cycles were associated with higher live birth rates and lower risks of miscarriage and LGA infants in singleton pregnancies. However, this protocol is associated with an increased risk of GDM. These findings provide valuable evidence for individualizing endometrial preparation protocols in PCOS patients undergoing FET, balancing efficacy and maternal safety.

**Supplementary Information:**

The online version contains supplementary material available at https://doi.org/10.1186/s12884-026-09640-x.

## Background

Polycystic ovary syndrome (PCOS), the most prevalent cause of anovulatory infertility, affects approximately 5% to 20% of women worldwide during their reproductive years [1]. In vitro fertilization and embryo transfer (IVF-ET) is an effective treatment for women with PCOS who experience recurrent ovulation induction failures or have additional infertility factors, such as tubal or male-related issues. However, PCOS patients undergoing IVF face a higher risk of ovarian hyperstimulation syndrome (OHSS). To mitigate this risk, a freeze-all strategy is often employed, making frozen embryo transfer (FET) a standard approach for PCOS patients in IVF treatment.

Unlike the complex ovarian stimulation protocols used in IVF to promote multifollicular growth, FET employs simpler regimens aimed at optimizing endometrial receptivity for thawed embryos. Although various cycle protocols have been developed to enhance the uterine environment for implantation, no single endometrial preparation method has demonstrated superior reproductive outcomes over others to date.

For endometrial preparation in women undergoing FET, various cycle regimens are employed, including natural cycles, ovulation induction cycles, and artificial cycles (AC). However, natural cycles are seldom used in PCOS patients due to oligo-anovulation and irregular menstruation. In contrast, AC—which establishes a controlled endometrial environment through exogenous estradiol and progesterone—is widely preferred for its scheduling flexibility and lower cycle cancellation rates. Ovulation induction cycles, which stimulate follicular growth using ovulation induction drugs, aim to replicate natural ovulation, but are less commonly adopted due to the need for frequent monitoring via ultrasound and hormonal assessments.

Current evidence regarding the impact of different endometrial preparation methods on maternal and neonatal outcomes in PCOS patients remains both limited and inconsistent. In a large retrospective analysis, PCOS patients undergoing letrozole-based FET cycles demonstrated significantly reduced rates of hypertensive disorders of pregnancy relative to those receiving AC [2, 3]. A cohort study comparing endometrial preparation protocols in PCOS patients undergoing freeze-all FET demonstrated significantly improved live birth rates with natural cycle and ovarian stimulation approaches versus AC, with no significant differences in obstetric or neonatal outcomes across protocols [4]. However, limited evidence exists regarding whether ovulation induction during FET cycles improves pregnancy outcomes in PCOS patients, and no large-scale studies have comprehensively evaluated both reproductive and maternal-neonatal outcomes.

To address this knowledge gap, we conducted a large retrospective cohort study with propensity score matching (PSM) to compare clinical, maternal, and perinatal outcomes between ovulation induction cycles and artificial cycles in PCOS women undergoing FET. Our study aims to provide evidence-based guidance for individualizing endometrial preparation protocols, balancing reproductive efficacy and maternal safety.

## Methods

### Study design and population

This retrospective cohort study was conducted at the Department of Assisted Reproduction, Northwest Women’s and Children’s Hospital in China, utilizing clinical data from January 2018 to December 2023. PCOS diagnosis was established per Rotterdam criteria [5], requiring at least two of the following: (1) oligo/anovulation, (2) clinical/biochemical hyperandrogenism, or (3) polycystic ovarian morphology (≥ 12 follicles [2–9 mm] or ovarian volume ≥ 10 cm³ per ovary). Inclusion criteria required: (1) confirmed PCOS diagnosis, (2) first FET cycle, and (3) use of either ovulation induction or artificial cycle endometrial preparation. Women utilizing donor oocyte-derived embryos were excluded from the study. The study protocol received ethical approval from the institutional review board of Northwest Women’s and Children’s Hospital (Approval No. 2023003). All participants underwent standardized clinical follow-up for 12 months post-embryo transfer. A standard 75-g oral glucose tolerance test (OGTT) is mandatory for all pregnant women between 24 and 28 weeks of gestation, with 100% screening coverage across both cohorts.

### Endometrial preparation

The choice between ovulation induction and AC for endometrial preparation in FET cycles was primarily determined by patient characteristics and physician discretion. In artificial cycle preparations, participants received standardized sequential estrogen-progesterone therapy. Oral estradiol valerate (Progynova^®^, Bayer) was initiated on menstrual cycle day 5. Following 10–12 days of estrogen priming, endometrial thickness was assessed via transvaginal ultrasound with confirmation of absent dominant follicle development. Progesterone supplementation commenced upon meeting dual criteria: (1) endometrial thickness ≥ 7 mm and (2) serum progesterone < 1.5 ng/mL. Progesterone was supplemented through both oral (dydrogesterone 40 mg/day; Duphaston, Solvay, Netherlands) and vaginal routes (micronized progesterone 200 mg/day; Utrogestan, Besins Manufacturing, Belgium), combined with oral estradiol. Blastocyst transfer was performed five days after starting progesterone administration.

For ovulation induction, 5 mg/day of letrozole (Femara, Novartis, Switzerland) was given orally for 5 days, beginning on cycle day 3 of spontaneous menses or progesterone-induced withdrawal bleeding. Ultrasound monitoring and serum hormone analysis were initiated on cycle day 10. If the leading follicle measured ≥ 14 mm on day 10, transvaginal ultrasound was repeated every 48 h, with no additional medications administered until ovulation triggering. However, if the dominant follicle remained < 14 mm on day 10, human menopausal gonadotropin (hMG, Livzon) was added at a daily dose of 75 IU to promote follicular growth, with potential incremental adjustments of 75 IU as needed. The patients were monitored daily with serum LH measurements until an LH surge (defined as LH ≥ 20 IU/L) was detected. Luteal phase support was initiated two days after the LH surge, using vaginal progesterone (micronized progesterone 200 mg/day; Utrogestan, Besins Manufacturing, Belgium). Embryo transfer was scheduled for 6 days post-LH surge. On the transfer day, frozen embryos were thawed and cultured for 1–2 h prior to transfer to ensure proper re-expansion.

Luteal phase support was maintained until 10 weeks of gestation. Clinical pregnancy was confirmed by transvaginal ultrasound through visualization of a gestational sac with fetal cardiac activity. The follow-up data—including perinatal complications, gestational age at delivery, birth date, mode of delivery, neonatal sex, and birth weight is routinely updated in real time by trained nurses and clinicians as part of standard clinical care.

### Outcome measures

The primary outcome was the live birth rate (LBR), defined as the delivery of one or more live-born infants after 24 weeks of gestation. Secondary outcomes included clinical outcomes as well as maternal and perinatal outcomes. For this study, we defined biochemical pregnancy as serum hCG > 50 IU/L at 14 days post-embryo transfer; clinical pregnancy as ultrasound detection of ≥ 1 gestational sac at 5 weeks; ongoing pregnancy as viability beyond 12 weeks; miscarriage as clinical pregnancy loss before 24 weeks, in accordance with WHO and UK guidelines; and ectopic pregnancy as extrauterine gestational sac on ultrasound. Additional definitions included: preterm birth (< 37 weeks); stillbirth (fetal death ≥ 24 weeks); GDM (OGTT meeting ≥ 1 threshold: fasting ≥ 5.1 mmol/L, 1-hour ≥ 10.0 mmol/L, or 2-hour ≥ 8.5 mmol/L) [6]; pregnancy-induced hypertension (SBP > 140 and/or DBP > 90 mmHg); and pre-eclampsia (new-onset hypertension with proteinuria).

### Statistical analysis

Continuous variables were first assessed for normality using the Shapiro–Wilk test. Variables following a normal distribution were presented as mean ± standard deviation (SD) and compared between groups using Student’s t-test. Non-normally distributed variables were presented as median with interquartile range (IQR, 25th–75th percentiles) and compared using the Mann–Whitney U test. Categorical variables were presented as counts and percentages. For comparisons of categorical variables, the chi-squared test was applied when all expected cell frequencies were ≥ 5; otherwise, Fisher’s exact test was used.

To address the imbalance in sample size between the two groups (artificial cycle: *n* = 2,722; ovulation induction: *n* = 543), propensity score matching (PSM) was performed using logistic regression. The propensity score model included the following baseline covariates: female age at embryo transfer, endometrial thickness on the day of progesterone administration, and body mass index (BMI). Ovulation induction patients were matched 1:1 to artificial cycle patients using nearest-neighbor matching with a caliper width of 0.1 standard deviation of the logit of the propensity score. Covariate balance between the matched groups was assessed using standardized differences (SD), with an absolute SD < 10% considered indicative of good balance. We constructed two multivariable models with sequential adjustment for potential confounders: Model I was adjusted for female age at embryo transfer, female age at oocyte retrieval, antral follicle count (AFC), and BMI. Model II was additionally adjusted for infertility duration, infertility type, type of embryo transferred (cleavage vs. blastocyst), number of good-quality embryos transferred, number of embryos transferred, endometrial thickness on the day of progesterone administration, triple-line endometrial pattern, and fertilization type, on top of the covariates included in Model I.

All clinical, maternal, and perinatal outcomes were compared between the matched pairs. Statistical analyses were performed using R software (version 3.4.3; The R Foundation) and Empower (R) (X&Y Solutions, Inc., Boston, MA). All tests were two-sided, and a P-value < 0.05 was considered statistically significant.

## Results

### Baseline characteristics

**Fig. 1:**
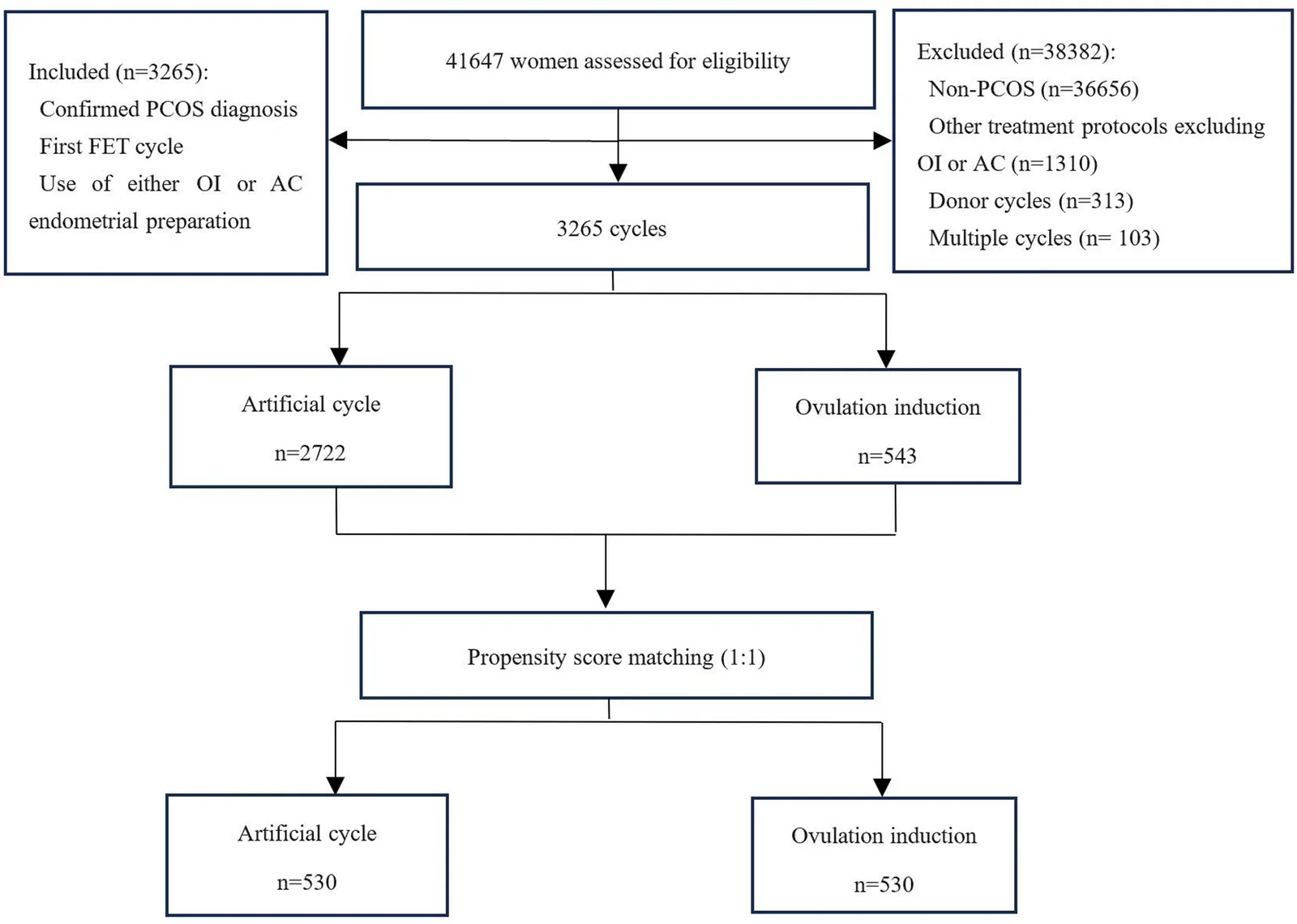
Flowchart of the study

Figure 1 illustrates the enrollment of eligible patients who completed follow-up without attrition after FET. In total, 3,255 women who had undergone FET were eligible. Among them, 2,722 women received artificial cycle preparation, and 543 women underwent the ovulation induction protocol. Due to the imbalance in sample size between the two groups (ratio ~ 5:1), PSM was performed to improve comparability. After 1:1 matching, 530 patients from each group were included in the primary analysis (Fig. 1).

Baseline characteristics of the study population before and after PSM are presented in Table 1. After 1:1 propensity score matching, all baseline covariates were well balanced between the two groups, with all standardized differences below 10%, indicating good covariate balance. The matched cohort therefore consisted of 530 patients in each group with similar baseline characteristics, minimizing the potential for confounding by the measured covariates.

**Table 1:** Baseline characteristics of artificial cycle and ovulation induction during frozen-thawed embryo transfer before and after PSM

	Before PSM			After PSM		
	AC (*n* = 2722)	Ovulation induction (*n* = 543)	Standardized Difference (%)	AC (*n* = 530)	Ovulation induction (*n* = 530)	Standardized Difference (%)
Female age at oocyte retrieval (y)	29.3 ± 3.4	30.0 ± 3.4	2	29.9 ± 3.6	30.0 ± 3.4	1
Female age at embryo transfer (y)	29.9 ± 3.5	30.9 ± 3.5	5	30.7 ± 3.7	30.9 ± 3.5	9
Male age (y)	31.6 ± 3.8	32.5 ± 3.9	11	32.1 ± 3.9	32.5 ± 3.9	9
Infertility duration(y)	3.0 (2.0–5.0)	3.0 (2.0–5.0)	1	4.0 (2.0–5.0)	3.0 (2.0–5.0)	9
BMI (kg/m^2^)	23.9 (21.4–26.7)	24.2 (21.5–27.7)	1	24.2 (21.9–26.9)	24.1 (21.5–27.7)	1
Baseline hormonal profile						
FSH (IU/L)	5.9 (4.9–6.9)	5.9 (5.0-7.1)	4	5.9 (4.8-7.0)	5.8 (5.0-7.1)	9
LH (IU/L)	7.1 (4.5–11.4)	6.6 (4.3–10.4)	10	7.1 (4.6–11.4)	6.6 (4.3–10.4)	9
E_2_ (pg/mL)	39.6 (29.3–57.4)	35.4 (26.4–49.0)	1	38.0 (29.0–56.0)	35.5 (26.8–49.0)	0
Testosterone (ng/mL)	48.0 (24.1–66.0)	45.8 (25.2–62.6)	1	48.6 (26.9–65.9)	46.1 (25.2–62.7)	9
AFC	24.0 (22.0–24.0)	24.0 (22.0–24.0)	10	24.0 (22.0–24.0)	24.0 (22.0–24.0)	1
Gravidity			7			1
0	1838 (67.7%)	386 (71.2%)		359 (67.9%)	361 (68.2%)	
≥1	877 (32.3%)	156 (28.8%)		170 (32.1%)	168 (31.8%)	
Parity			17			1
0	2528 (93.3%)	517 (95.4%)		482 (91.1%)	483 (95.3%)	
≥1	182 (6.7%)	25 (4.6%)		47 (8.9%)	46 (4.7%)	
Infertility type, n (%)			7			9
Primary infertility	1841 (67.6%)	382 (70.5%)		358 (67.5%)	374 (70.7%)	
Secondary infertility	881 (32.4%)	160 (29.5%)		172 (32.5%)	155 (29.3%)	
Endometrial thickness on the day of progesterone administration	10.0 (9.0–11.0)	10.9 (9.5–12.1)	50	10.6 (9.5–11.9)	10.9 (9.5–12.0)	9
Triple-line endometrial pattern			13			1
A	324 (11.9%)	131 (24.1%)		124 (23.4%)	129 (24.3%)	
B	1567 (57.6%)	271 (49.9%)		273 (51.5%)	269 (50.8%)	
C	831 (30.5%)	141 (26.0%)		133 (25.1%)	132 (24.9%)	
Gonadotropin dosage in fresh cycles (IU)	1925.0 (1425.0-2525.0)	1800.0 (1350.0-2400.0)	10	1975.0 (1500.0-2612.5)	1800.0 (1347.9–2400.0)	9
Gonadotropin duration in fresh cycles (days)	11.0 (9.0–13.0)	10.0 (9.0–12.0)	20	11.0 (9.0–13.0)	10.8 (9.7–12.0)	8
Number of oocytes retrieved in fresh cycles (n)	18.0 (13.0–23.0)	17.0 (13.0–22.0)	10	17.0 (13.0–22.0)	17.0 (13.0–22.0)	1
Fertilization type, n (%)			3			1
IVF	2209 (81.2%)	460 (84.7%)		458 (86.4%)	452 (85.3%)	
ICSI	448 (16.5%)	74 (13.6%)		65 (12.3%)	69 (13.0%)	
IVF+ICSI	65 (2.4%)	9 (1.7%)		7 (1.3%)	9 (1.7%)	
Number of embryos available	9.0 (6.0–12.0)	8.0 (6.0–12.0)	10	8.0 (6.0–12.0)	8.0 (6.0–12.0)	1
Number of embryos transferred, n (%)			1			9
1	384 (14.1%)	70 (12.9%)		417 (78.8%)	437 (82.6%)	
2	2330 (85.9%)	472 (87.1%)		112 (21.2%)	92 (17.4%)	
Number of good quality embryos transferred			1			1
0	1091 (40.1%)	212 (39.0%)		219 (41.3%)	207 (39.1%)	
1	1481 (54.5%)	297 (54.7%)		280 (52.8%)	289 (54.5%)	
2	147 (5.4%)	34 (6.3%)		31 (5.8%)	34 (6.4%)	
Type of embryo transferred, n (%)			5			1
Cleavage stage	384 (14.1%)	70 (12.9%)		77 (14.6%)	68 (12.9%)	
Blastocyst stage	2330 (85.9%)	472 (87.1%)		452 (85.4%)	461 (87.1%)	

Unless stated otherwise values are mean ± standard deviation

*AC* artificial cycle, *BMI* body mass index, *FSH* follicle-stimulating hormone, *LH* luteinizing hormone, *E*_*2*_ estradiol, *AFC* antral follicle count, *PCOS* polycystic ovarian syndrome, *IVF* in vitro fertilization, *ICSI* intracytoplasmic sperm injection

### Clinical outcomes between ovulation induction and AC

The clinical outcomes of the ovulation induction and AC are presented in Table 2. After multivariable adjustment for potential confounders, the results remained largely consistent. In Model I, which adjusted for female age at embryo transfer, female age at oocyte retrieval, AFC, and BMI, the ovulation induction group demonstrated a significantly higher live birth rate (aOR 1.3, 95% CI 1.0–1.6) and a significantly lower miscarriage rate (aOR 0.7, 95% CI 0.5–1.0) compared with the AC group. In Model II, which additionally adjusted for infertility duration, infertility type, type of embryo transferred (cleavage vs. blastocyst), number of good-quality embryos transferred, number of embryos transferred, endometrial thickness on the day of progesterone administration, triple-line endometrial pattern, and fertilization type, the beneficial effects of ovulation induction remained significant for live birth (aOR 1.3, 95% CI 1.0–1.7) and miscarriage (aOR 0.6, 95% CI 0.4–0.9). No statistically significant differences were observed between the two groups for biochemical pregnancy, clinical pregnancy, ongoing pregnancy, or ectopic pregnancy in either model.

**Table 2:** Clinical outcomes comparing ovulation induction versus artificial cycle in frozen-thawed embryo transfer after propensity score matching

Characteristic	AC (*n* = 530)	Ovulation induction (*n* = 530)	Model Ⅰ OR (95%CI)^a^	Model Ⅱ OR (95%CI)^b^
Biochemical pregnancy	388 (73.3%)	389 (73.5%)	1.0 (0.8, 1.3)	1.0 (0.7, 1.3)
Clinical pregnancy	358 (67.7%)	370 (69.9%)	1.1 (0.9, 1.4)	1.1 (0.8, 1.4)
Ongoing pregnancy	346 (65.4%)	364 (68.8%)	1.2 (0.9, 1.5)	1.2 (0.9, 1.5)
Miscarriage	84 (15.8%)	59 (11.1%)	**0.7 (0.5**,** 1.0)**	**0.6 (0.4**,** 0.9)**
Ectopic pregnancy	2 (0.4%)	5 (0.9%)	2.5 (0.5, 13.0)	2.6 (0.5, 14.3)
Live birth	272 (51.3%)	305 (57.5%)	**1.3 (1.0**,** 1.6)**	**1.3 (1.0**,** 1.7)**

Model Ⅰ: Analyses were adjusted for female age at embryo transfer, female age at oocyte retrieval, AFC, and BMI

Model Ⅱ: Analyses were adjusted for female age at embryo transfer, AFC, BMI, infertility duration, infertility type, type of embryo transferred (cleavage vs. blastocyst), number of good quality embryos transferred, number of embryos transferred, endometrial thickness on the day of progesterone administration, triple-line endometrial pattern, and fertilization type

*AC* artificial cycle, *CI* confidence interval, *OR* odds ratio

Values in bold indicate statistically significant differences

Among the 530 patients in the ovulation induction group after PSM, 139 received letrozole alone and 391 required additional hMG supplementation (letrozole + hMG). As shown in Supplementary Table 1, no statistically significant differences were observed between the two subgroups in any clinical outcome (biochemical pregnancy, clinical pregnancy, ongoing pregnancy, miscarriage, ectopic pregnancy, or live birth) after adjustment for potential confounders in Model I and Model II.

### Maternal and perinatal outcomes

In ovulation induction cycles, rates of gestational diabetes mellitus were higher compared with AC (12.0% vs. 7.9%; aOR 1.5, 95% CI 1.1–2.1) (Table 3). In addition, no difference in hypertensive disorders of pregnancy, antepartum haemorrhage, preterm birth, preterm premature rupture of membranes (PPROM), mode of delivery, cesarean section, gestational age at birth, and stillbirth were reported between the ovulation induction group and AC group. A further analysis compared fetal outcomes between ovulation induction and AC in singleton and twin populations separately. Infants birth weights, incidence of low birth weight, very low birth weight, high birth weight, very high birth weight, large for gestation age (LGA), small for gestation age (SGA), and congenital anomaly were not significantly different between the two groups except for LGA in singleton. Among singletons, LGA rates were lower in ovulation induction cycles compared with AC (22.6% vs. 26.8%; aOR 0.7, 95% CI 0.5–0.9).

**Table 3:** Maternal and perinatal outcomes between artificial cycle and ovulation induction in ongoing pregnancies after propensity score matching

Clinical outcomes	AC			Ovulation induction		OR/Mean Difference (95%CI)	*P* value
	*N*		*n*(%)/median(Q1-Q3)	*N*	*n*(%)/median(Q1-Q3)		
Gestational diabetes mellitus	346		31 (9.0%)	364	64 (17.6%)	**2.2 (1.4**,** 3.4)**	**< 0.01**
Hypertensive disorders of pregnancy	346		27 (7.8%)	364	35 (9.6%)	1.3 (0.7, 2.1)	0.39
Pregnancy-induced hypertension	346		26 (7.5%)	364	34 (9.3%)	1.3 (0.7, 2.2)	0.38
Pre-eclampsia	346		1 (0.3%)	364	1 (0.3%)	1.0 (0.1, 15.3)	0.97
Antepartum haemorrhage	346		1 (0.3%)	364	3 (0.8%)	2.9 (0.3, 27.7)	0.34
Placenta previa	346		1 (0.3%)	364	2 (0.5%)	1.9 (0.2, 21.1)	0.59
Placenta accreta	346		0 (0.0%)	364	1 (0.3%)	―	0.33
Preterm birth	346		49 (14.2%)	364	52 (14.3%)	1.0 (0.7, 1.5)	0.96
PPROM	346		7 (2.0%)	364	9 (2.5%)	1.2 (0.5, 3.3)	0.69
Mode of delivery, cesarean section	346		207 (59.8%)	364	225 (61.8%)	1.1 (0.8, 1.5)	0.59
Gestational age at birth (weeks)	346		38.3 (2.3)	364	38.2 (1.9)	-0.1 (-0.3, 0.2)	0.47
Stillbirth	346		0 (0.0%)	364	0 (0.0%)	―	―
Singleton							
Birth weight (g)	249		3450.0 (3100.0-3770.0)	282	3250.0 (2955.0-3600.0)	**-170.5 (-263.7**,** -77.3)**	**< 0.01**
Low birth weight (< 2500 g)	249		15 (6.0%)	282	25 (8.9%)	1.5 (0.8, 2.9)	0.22
Very low birth weight (< 1500 g)	249	1 (0.4%)		282	2 (0.7%)	1.8 (0.2, 19.7)	0.64
High birth weight (> 4000 g)	249	25 (10.0%)		282	19 (6.7%)	0.6 (0.3, 1.2)	0.17
Very high birth weight (> 4500 g)	249	3 (1.2%)		282	1 (0.4%)	0.3 (0.0, 2.8)	0.26
Large for gestational age	249	92 (36.9%)		282	64 (22.7%)	**0.5 (0.3**,** 0.7)**	**< 0.01**
Small for gestational age	249	8 (3.2%)		282	6 (2.1%)	0.7 (0.2, 1.9)	0.44
Congenital anomaly	249	4 (1.6%)		282	3 (1.1%)	0.7 (0.1, 3.0)	0.58
Twins							
Birth weight (g)	23	2405.0 (2062.5-2687.5)		23	2430.0 (2255.0-2717.5)	97.2 (-182.3, 376.6)	0.50
Low birth weight (< 2500 g)	23	15 (65.2%)		23	13 (56.5%)	0.7 (0.2, 2.3)	0.55
Very low birth weight (< 1500 g)	23	1 (4.3%)		23	1 (4.3%)	1.0 (0.1, 17.0)	0.99
High birth weight (> 4000 g)	23	0 (0.0)		23	0 (0.0)	―	―
Very high birth weight (> 4500 g)	23	0 (0.0)		23	0 (0.0)	―	―
Large for gestational age	23	1 (4.3%)		23	0 (0.0)	―	―
Small for gestational age	23	0 (0.0%)		23	1 (7.1%)	―	―
Congenital anomaly	23	0 (0.0)		23	0 (0.0)	―	―

*AC* artificial cycle, *PPROM* preterm premature rupture of membranes, *CI* confidence interval, *OR* odds ratio

Values in bold indicate statistically significant differences

## Discussion

In this propensity score-matched cohort study of PCOS women undergoing FET, we found that ovulation induction for endometrial preparation was associated with a significantly higher live birth rate and a lower miscarriage rate compared with AC, albeit at the cost of an increased risk of GDM. These associations persisted after rigorous adjustment for potential confounders. However, given the retrospective design and the inherent trade-off between reproductive efficacy and metabolic safety, these findings should be interpreted with caution and weighed against individual patient profiles.

In recent years, growing concerns have emerged regarding whether assisted reproductive technology (ART) exerts adverse effects on maternal and offspring health. PCOS, characterized by metabolic and endocrine dysregulations, has been linked to elevated risks of GDM, preeclampsia, preterm birth, and LGA infants [7–9]. Thus, the development of a safer and more effective FET regimen tailored for PCOS patients is imperative.

For many years, the AC protocol has served as the standard of care for endometrial preparation in anovulatory women undergoing FET cycles. This approach is primarily favored for its scheduling flexibility, which allows for convenient embryo transfer date arrangements and effectively avoids weekend procedures, along with previously documented lower cycle cancellation rates. However, AC is not without limitations: they necessitate prolonged progesterone injections—administered until the placenta assumes full hormone production by the 10th week of gestation—entailing both increased injection frequency and duration.

Letrozole differs from clomiphene citrate as it isn’t an endometrial estrogen antagonist, thus avoiding thin lining. It’s ideal for ovulation induction and endometrial preparation in anovulatory FET patients [10]. While gonadotropins and hCG are alternatives with similar pregnancy rates to programmed cycles, letrozole offers advantages: oral administration, lower cost, reduced risk of multifollicular development and ovarian hyperstimulation syndrome (OHSS), and better tolerability than injectables. These benefits make letrozole FET protocols the preferred choice for this population.

A previous meta-analysis has shown that stimulated protocols yielded significantly higher live birth rates compared to AC among PCOS women undergoing FET [11], which aligns with the conclusions of our study. Substantial evidence indicates that the corpus luteum has multifaceted roles in early pregnancy beyond producing estradiol and progesterone, including the secretion of relaxin and vascular endothelial growth factor (VEGF) [12, 13]. It has been hypothesized that the deficiency of the vasoactive and angiogenic growth factors, resulting from the absence of a corpus luteum during the early stages of pregnancy, may give rise to abnormal implantation and placentation, which in turn could lead to miscarriage [14–16]. Consistent with this observation, our findings demonstrated a significantly lower miscarriage rate in ovulation induction cycles compared to those utilizing AC.

Interestingly, our study revealed a significantly higher GDM rate in ovulation induction cycles compared to AC. This finding is consistent with the observations reported by Yuan Y et al. [16]. This result aligns with a large cohort study by Saito et al. [17], which demonstrated that reduced secretion of insulin-counteracting hormones such as progesterone and human placental lactogen decreases the risk of GDM in pregnancies conceived via artificial cycle FET. While GDM is known to be associated with maternal characteristics such as age, obesity, family history of diabetes, and ethnicity, our findings indicate that assisted reproductive technology treatment also influences the risk of GDM. In this study, the increased incidence of placenta-related diseases in patients who underwent AC suggests that the risk of GDM may also be modulated by anatomical or functional placental changes. Specifically, reduced secretion of insulin-counteracting hormones from the placenta might suppress GDM pathogenesis in certain AC pregnancies. However, since this study did not investigate hormonal status or placental characteristics, further research is warranted to clarify the potential association between peri-implantation period hormonal profiles and subsequent GDM development.

PCOS upregulates androgen receptor expression (such as Cyr61) while downregulating integrin αvβ3 expression [18]. Integrins are thought to mediate the initial attachment of embryos to the endometrium, and reduced integrin expression during the luteal phase of the menstrual cycle is hypothesized to impair blastocyst implantation [19, 20]. Inadequate embryo implantation and shallow cytotrophoblast invasion may lead to incomplete spiral artery remodeling, contributing to the pathogenesis of preeclampsia [14]. Ovulation induction with optimized hormone levels and enhanced endometrial receptivity may facilitate decidualization, promote extravillous trophoblast cell invasion, and enhance placentation. These mechanisms may explain the reduced incidence of LGA infants in ovulation induction cycles.

For clinical practice, the findings offer actionable guidance for endometrial preparation in PCOS women undergoing FET. Given the higher live birth rate and lower miscarriage risk with ovulation induction compared to AC, clinicians may prioritize this strategy for PCOS patients seeking to optimize reproductive outcomes—especially those with a history of pregnancy loss or suboptimal FET success. However, this preference must be balanced with proactive monitoring for GDM: since ovulation induction increases GDM risk, early screening (e.g., glucose tolerance tests) and lifestyle interventions should be integrated into prenatal care for these patients. Additionally, the lower prevalence of LGA infants with ovulation induction may reduce neonatal complications, further support its clinical utility when paired with GDM prevention.

For research, the study highlights critical gaps requiring further investigation. First, mechanistic studies are needed to clarify how different endometrial preparation strategies influence obstetrical outcomes—particularly the link between ovulation induction, corpus luteum function, and GDM development. Second, long-term follow-up studies should assess whether the metabolic risks associated with ovulation induction translate to lasting maternal or child health impacts. Third, comparative effectiveness research could explore personalized approaches: identifying subgroups of PCOS patients who may benefit most from ovulation induction to balance reproductive efficacy and metabolic safety. Finally, studies evaluating interventions to mitigate GDM risk in ovulation induction cycles would address the key trade-off identified in this research.

Our findings offer several actionable insights for clinical practice. First, for PCOS patients undergoing FET, ovulation induction may be prioritized over AC to optimize live birth rates and reduce miscarriage risk. Second, the increased GDM risk associated with ovulation induction underscores the need for early and enhanced prenatal screening (e.g., oral glucose tolerance testing at 24–28 weeks’ gestation, or earlier in high-risk patients) and lifestyle interventions (e.g., dietary counseling, weight management) in this population. Third, the lower prevalence of large-for-gestational-age infants with ovulation induction may translate to reduced neonatal metabolic complications, further supporting its clinical utility. However, the choice between ovulation induction and AC should be individualized, weighing the reproductive benefits against the metabolic risks, and incorporating patient preferences, logistical considerations, and institutional expertise.

Several directions for future research are warranted. First, large-scale, multicenter prospective studies or randomized controlled trials (RCTs) are needed to confirm our findings and to establish causality. Second, mechanistic studies exploring the endocrinological and immunological differences between ovulation induction and AC cycles—particularly the role of corpus luteum-derived factors—are essential to elucidate the biological pathways underlying the observed differences in miscarriage and GDM rates. Third, subgroup analyses based on patient characteristics may help identify which patients derive the greatest reproductive benefit from ovulation induction while minimizing metabolic risks. Fourth, cost-effectiveness analyses comparing the two protocols are warranted to inform healthcare resource allocation and patient counseling.

### Strengths and limitations

The primary strengths of this study include its large cohort size and robust evidence demonstrating an association between endometrial preparation regimens and maternal/perinatal complications in frozen embryo transfer cycles among women with PCOS. Additionally, as a single-center study, the standardized endometrial preparation techniques across all groups ensured consistent protocol application and appropriate patient allocation.

Several limitations of this study should be acknowledged. First, this was a retrospective cohort study, which is inherently subject to selection bias and information bias. Although we employed PSM and multivariable regression to minimize confounding, the possibility of residual confounding due to unmeasured variables cannot be excluded. Second, despite PSM achieving balance in measured covariates, there was substantial group imbalance prior to matching (AC: *n* = 2,722; ovulation induction: *n* = 543), which may limit the representativeness of the matched cohort, as many AC patients were excluded. Third, this was a single-center study, and the results may not be generalizable to other centers with different patient populations, clinical practices, or laboratory conditions. Fourth, the relatively small number of events for certain outcomes—particularly ectopic pregnancy and LGA—precluded more detailed subgroup or stratified analyses. Fifth, the post-hoc power analysis revealed that while the study was adequately powered to detect the observed difference in live birth rate (achieved power: 82.3%), the power for miscarriage rate was slightly below the conventional threshold (achieved power: 76.5%), suggesting that the miscarriage finding should be interpreted with some caution and warrants validation in larger cohorts. Finally, although baseline metabolic parameters (e.g., HOMA-IR, fasting glucose) were not included as covariates in our regression models, all patients in this study had undergone standardized pre-IVF metabolic screening and were deemed metabolically normal prior to FET. This inclusion criterion minimized baseline metabolic heterogeneity between the two groups. However, we acknowledge that we did not perform a formal comparison of these parameters between groups, and the possibility of subtle residual metabolic differences—particularly between PCOS phenotypes—cannot be completely excluded. We acknowledge that fasting glucose, HOMA-IR, raw OGTT values and GDM severity classification were not available in this study, which precluded analyses of the relationship between endometrial preparation protocols and the degree of glucose intolerance. Future studies incorporating detailed OGTT data and GDM severity grading are warranted to further explore these associations. We acknowledge that treatment allocation was influenced by patient characteristics and physician discretion rather than randomization, which introduces potential selection bias. Although we employed propensity score matching to address measured and unmeasured confounding, the possibility of residual selection bias cannot be completely excluded.

A notable paradox in our findings requires explicit discussion: the ovulation induction group had a significantly higher GDM risk yet a lower LGA incidence. This counterintuitive pattern suggests that the underlying mechanisms may diverge from the classic GDM-LGA pathway. We postulate that the presence of a corpus luteum in OI cycles improves early placentation and reduces the risk of placenta-mediated fetal overgrowth, but this enhanced endocrine milieu may concurrently impose a higher metabolic load, increasing GDM susceptibility. Additionally, closer prenatal surveillance in the OI group—prompted by their higher baseline risk—may have led to earlier lifestyle interventions, potentially attenuating the progression from GDM to LGA. However, due to the lack of detailed data on GDM severity and intervention intensity, these hypotheses remain speculative. Future prospective studies integrating placental function assessment and metabolic phenotyping are warranted to validate this trade-off mechanism.

## Conclusions

In conclusion, this propensity score-matched retrospective cohort study demonstrates that among PCOS women undergoing FET, ovulation induction for endometrial preparation is associated with a higher live birth rate and a lower miscarriage rate compared with AC, albeit with a higher risk of GDM and a lower prevalence of LGA infants in singleton pregnancies. These findings suggest that ovulation induction may represent a viable and potentially preferable alternative to AC for reproductive outcomes in PCOS patients. However, these conclusions must be interpreted within the context of the study’s limitations, including its retrospective design, potential residual confounding, group imbalance prior to matching, and single-center setting. The trade-off between improved reproductive efficacy and increased metabolic risk underscores the need for individualized treatment decisions and highlights the importance of future prospective studies to optimize endometrial preparation strategies in this high-risk population.

## Supplementary Information

Supplementary Material 1.

## Data Availability

The datasets used and/or analysed during the current study are available from the corresponding author on reasonable request.
