# Supplementary material for: Ovulation induction versus artificial cycles in polycystic ovary syndrome: higher live birth rate but elevated gestational diabetes risk
Source: BMC Pregnancy Childbirth. 2026 Jul 14;26:1127. doi: 10.1186/s12884-026-09640-x (PMC13644214; doi:10.1186/s12884-026-09640-x)
Supplement: Supplementary file 1 [file 12884_2026_9640_MOESM1_ESM.docx]

**Supplementary TABLE 1**

**Clinical outcomes comparing letrozole only versus letrozole + hMG in frozen-thawed embryo transfer after propensity score matching**

| **Characteristic** | **Letrozole only (n=139)** | **Letrozole + hMG (n=391)** | **Model Ⅰ**  **OR (95%CI)^a^** | **Model Ⅱ**  **OR (95%CI)^b^** |
| --- | --- | --- | --- | --- |
| Biochemical pregnancy | 98 (70.5%) | 291 (74.6%) | 1.2 (0.8, 2.0) | 1.0 (0.5, 2.0) |
| Clinical pregnancy | 91 (65.5%) | 279 (71.5%) | 1.3 (0.8, 2.0) | 1.2 (0.7, 2.4) |
| Ongoing pregnancy | 90 (64.7%) | 274 (70.3%) | 1.3 (0.9, 2.1) | 1.5 (0.8, 2.8) |
| Miscarriage | 13 (9.4%) | 46 (11.8%) | 1.2 (0.6, 2.5) | 0.9 (0.4, 2.4) |
| Ectopic pregnancy | 1 (0.7%) | 4 (1.0%) | 1.0 (0.1, 10.2) | 1.8 (0.1, 46.6) |
| Live birth | 76 (54.7%) | 229 (58.6%) | 1.2 (0.8, 1.8) | 1.3 (0.7, 2.4) |

Model Ⅰ: Analyses were adjusted for female age at embryo transfer, female age at oocyte retrieval, AFC, and BMI.

Model Ⅱ: Analyses were adjusted for female age at embryo transfer, AFC, BMI, infertility duration, infertility type, type of embryo transferred (cleavage vs. blastocyst), number of good quality embryos transferred, number of embryos transferred, endometrial thickness on the day of progesterone administration, triple-line endometrial pattern, and fertilization type.

CI = confidence interval; OR = odds ratio.
